# The Use of Hydrobromic Ether in Labor

**Published:** 1880-03

**Authors:** Laurence Turnbull

**Affiliations:** Philadelphia


ARTICLE V.

### ON THE USE OF HYDROBROMIC ETHER IN LABOR.

BY LAURENCE TURNBULL, M. D., PHILADELPHIA.

In some instances, in labor, both ether and chloroform will produce, in certain females, so much relaxation of the sphincters that if there is the slightest tendency to hemorrhage it will be very much increased. To test the influence of hydrobromic ether, I administered it February 24, 1880, to Mrs. R. T. P——, aged 30, a lady with a narrow pelvic outlet and very rigid os uteri, who had been in labor with her fourth child for nine hours, having made but little progress. The hydrobromic ether was used in tablespoonful quantities, when the pains were most intense and distressing, and gave as prompt relief as ether, and yet it did not interfere in the least with the expulsive efforts. Her pulse was only increased six beats; no apparent disturbance of her respiration, only a feeling of fullness in the chest. The baby, a female, was born full of life and vigor, and cried lustily. There was a slight laceration of the perineum, as the occiput was pressed strongly upon the tissues. The patient had not a bad symptom from the use of this form of anæsthetic, and there was no sickness of the stomach.

The whole quantity employed was five tablespoonfuls. There was no hemorrhage, and the placenta was expelled with but slight traction. This is the first time that I am aware of this agent having been employed for this purpose.

